# Supplementary figures and images for: The visual white matter connecting human area prostriata and the thalamus is retinotopically organized
Source: Brain Struct Funct. 2020 Jun 13;225(6):1839–53. doi: 10.1007/s00429-020-02096-5 (PMC7321903; doi:10.1007/s00429-020-02096-5)

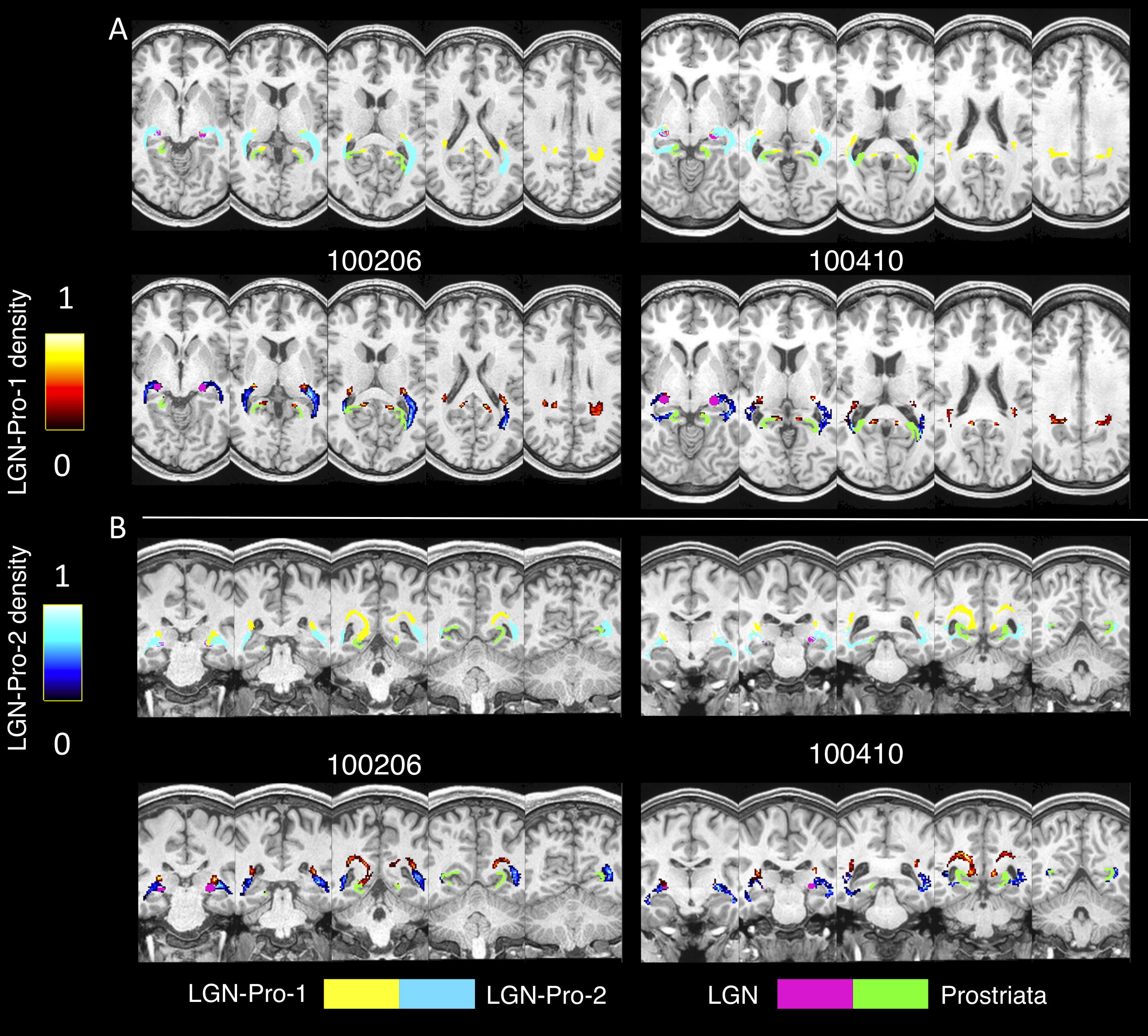

Supplement: Supplementary file 1 — Supplementary file1 (TIFF 21922 kb) [file 429_2020_2096_MOESM1_ESM.tiff]

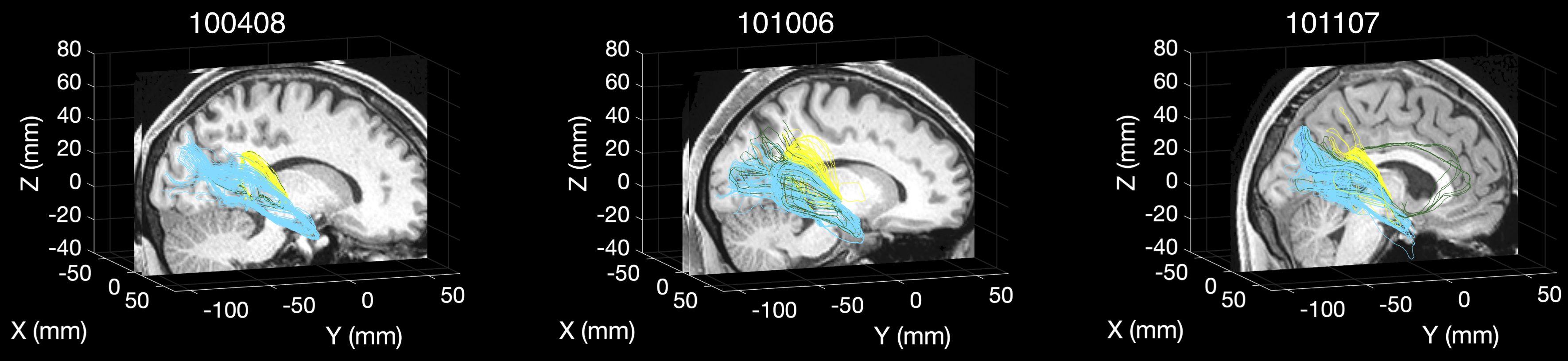

Supplement: Supplementary file 2 — Supplementary file2 (TIFF 11271 kb) [file 429_2020_2096_MOESM2_ESM.tiff]

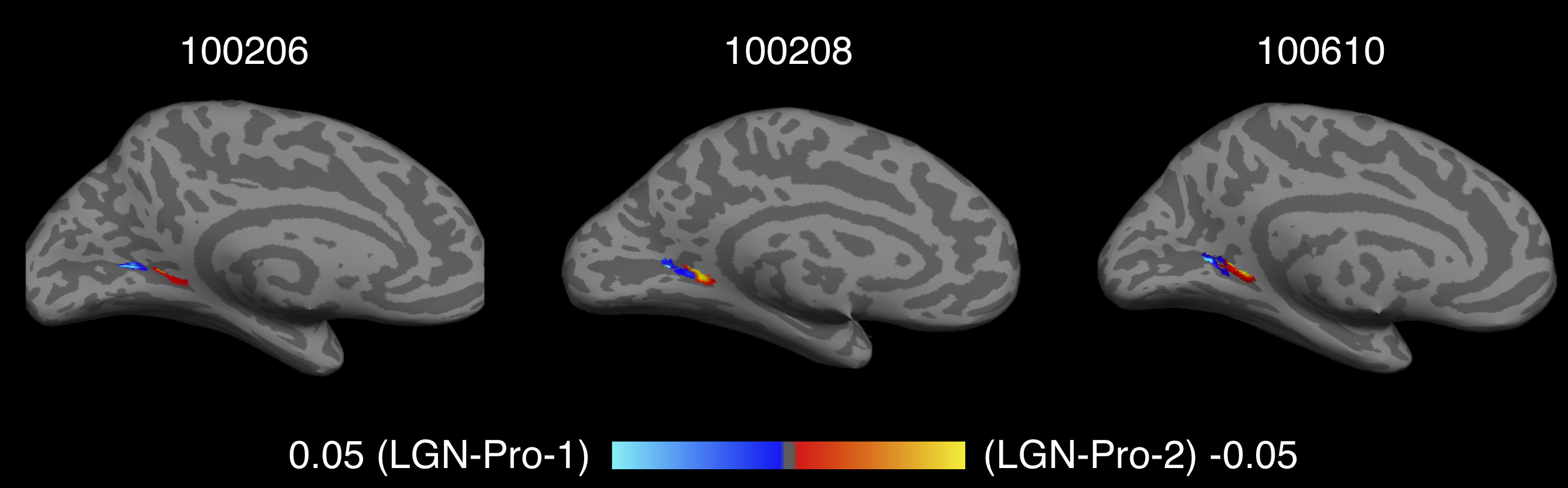

Supplement: Supplementary file 3 — Supplementary file3 (TIFF 8691 kb) [file 429_2020_2096_MOESM3_ESM.tiff]

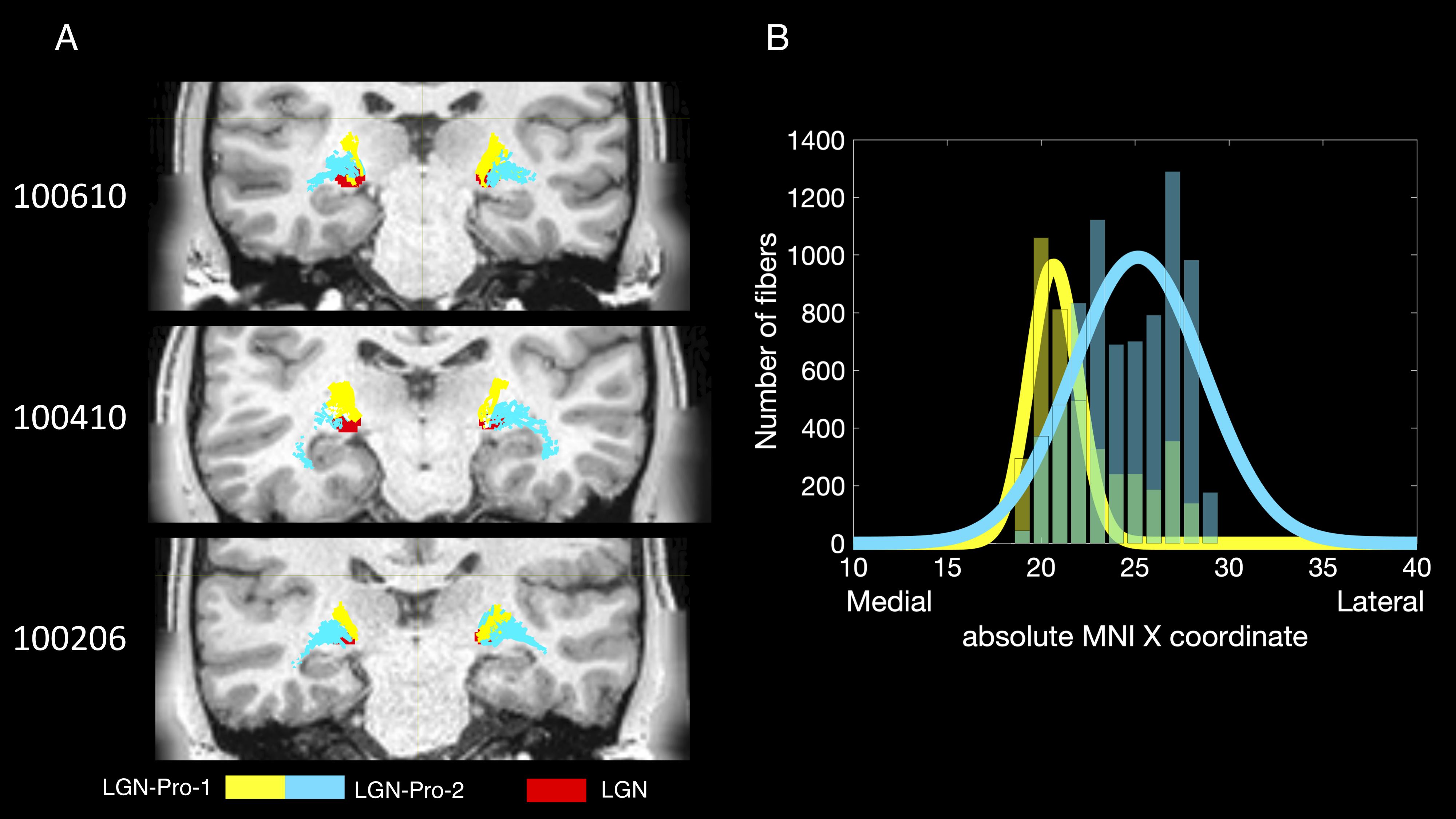

Supplement: Supplementary file 4 — Supplementary file4 (TIFF 35159 kb) [file 429_2020_2096_MOESM4_ESM.tiff]
