# Supplementary material for: The visual white matter connecting human area prostriata and the thalamus is retinotopically organized
Source: Brain Struct Funct. 2020 Jun 13;225(6):1839–53. doi: 10.1007/s00429-020-02096-5 (PMC7321903; doi:10.1007/s00429-020-02096-5)
Supplement: Supplementary file 5 — Supplementary file5 (DOCX 11873 kb) [file 429_2020_2096_MOESM5_ESM.docx]

**Electronic Supplementary Material**


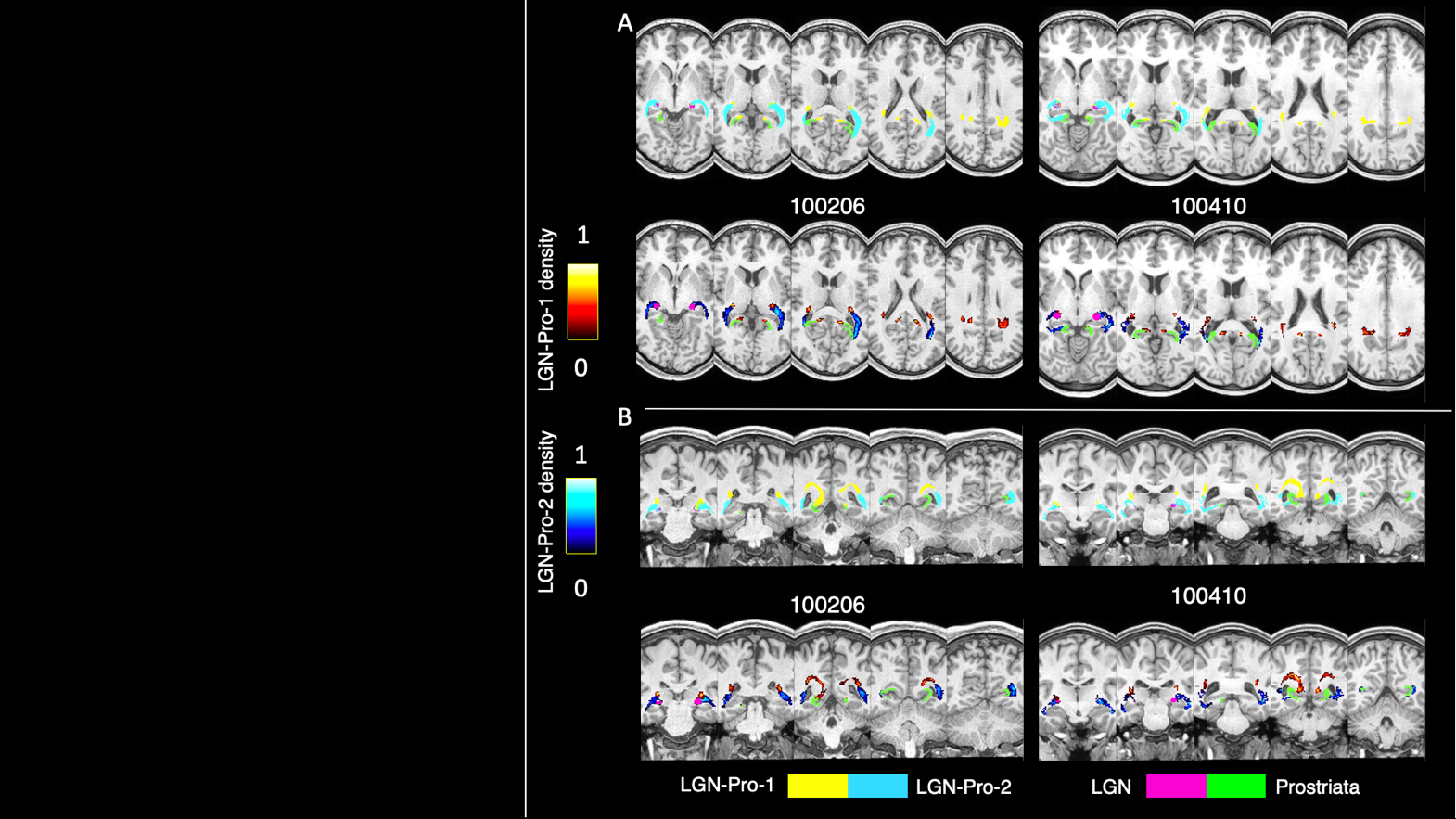


Supplementary Figure 1 - Slice by Slice tract profiles together with normalized density maps for two example subjects presented in coronal and axial planes. LGN and prostriata are outlined in pink and green colors

*
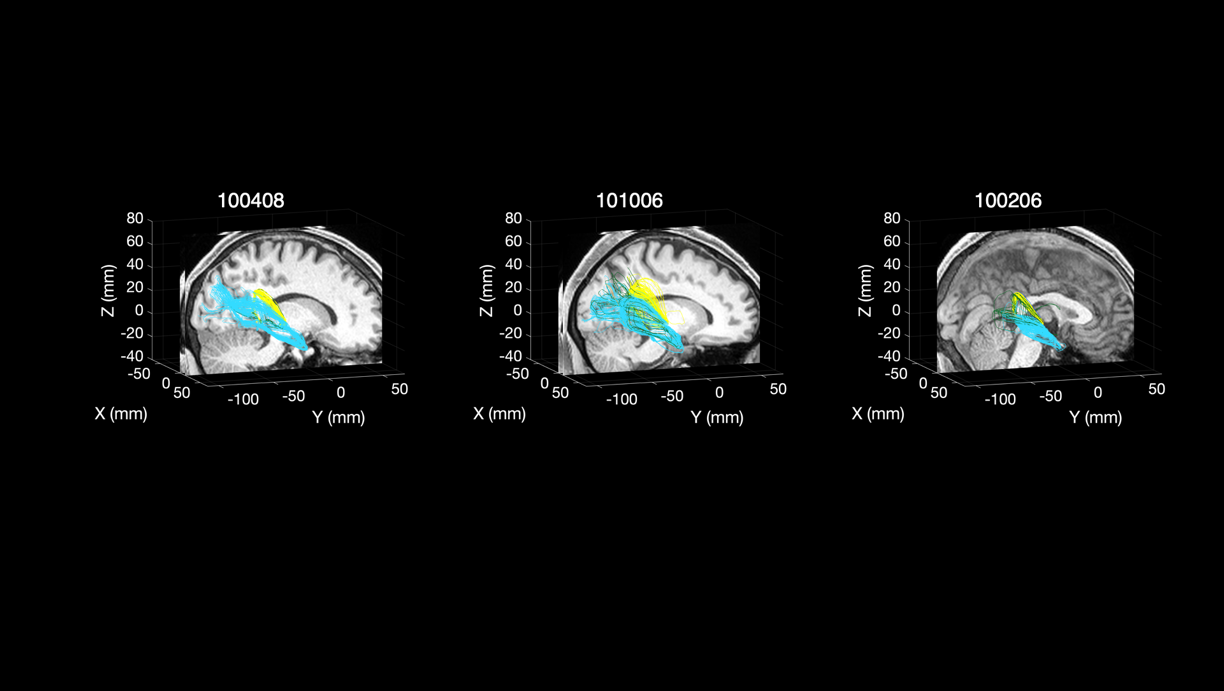
*

Supplementary Figure 2 – Results of the right hemispheres. Related to Figure 3A


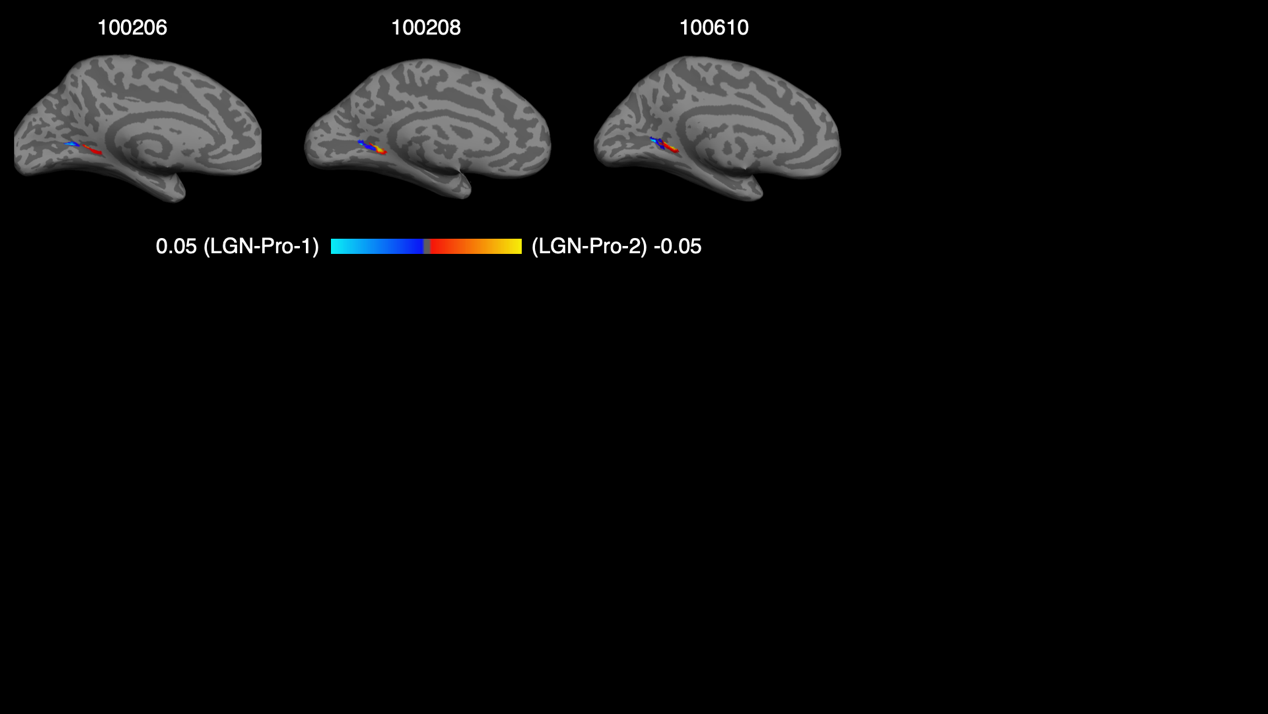


Supplementary Figure 3 – Results of the left hemispheres. Related to Figure 5A


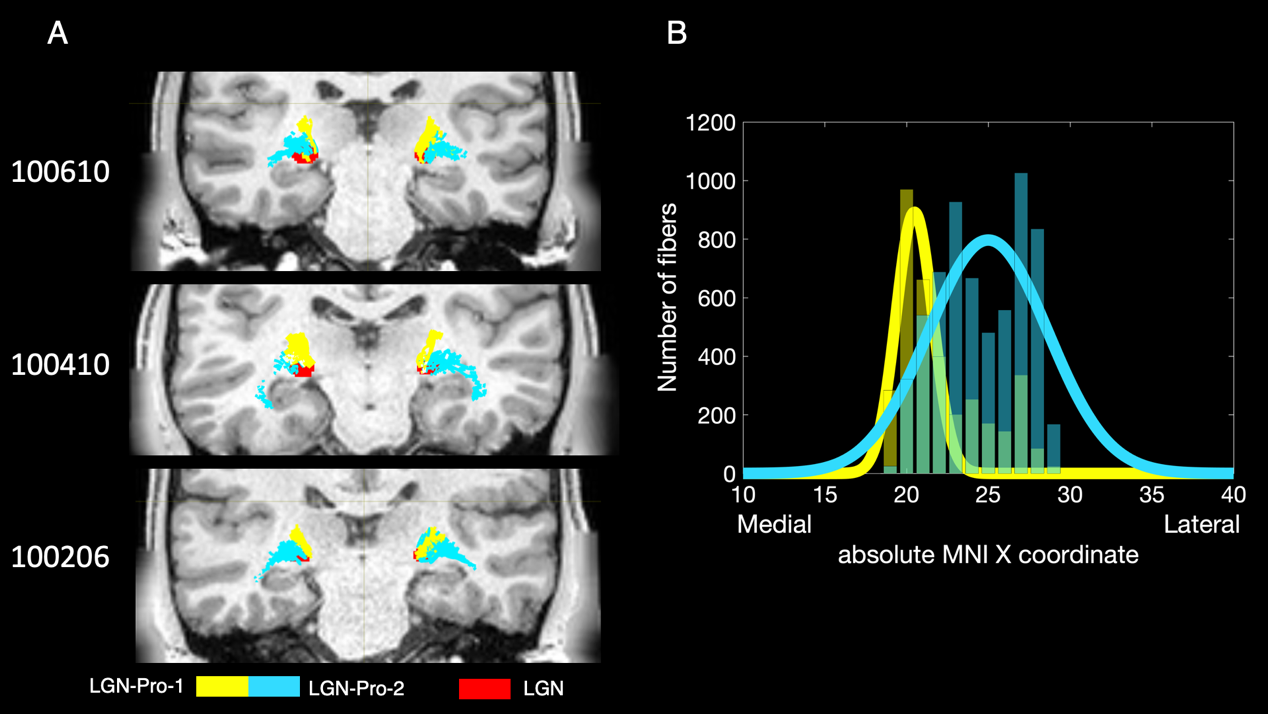


Supplementary Figure 4 - Three example subjects with a coronal slice positioned at the location of the LGN. LGN-Pro-1 enters the LGN from a superior direction reaching it medial parts while LGN-Pro-2 reaches LGN from the lateral side and finishes more laterally. B summarizes the findings as a group histogram (averaged across hemispheres) where both subcomponents are examined as a function of X coordinate in the MNI space. Thicker lines present a Gaussian fit on the binned data.
